# Supplementary material for: Synthesis of Starch-Based Ag2[Fe (CN)5NO] Nanoparticles for Utilization in Antibacterial and Wound-Dressing Applications
Source: Antioxidants (Basel). 2024 Jan 25;13(2):154. doi: 10.3390/antiox13020154 (PMC10886034; doi:10.3390/antiox13020154)
Supplement: Supplementary file 1 [file antioxidants-13-00154-s001.zip › antioxidants-2801047-supplementary.pdf]

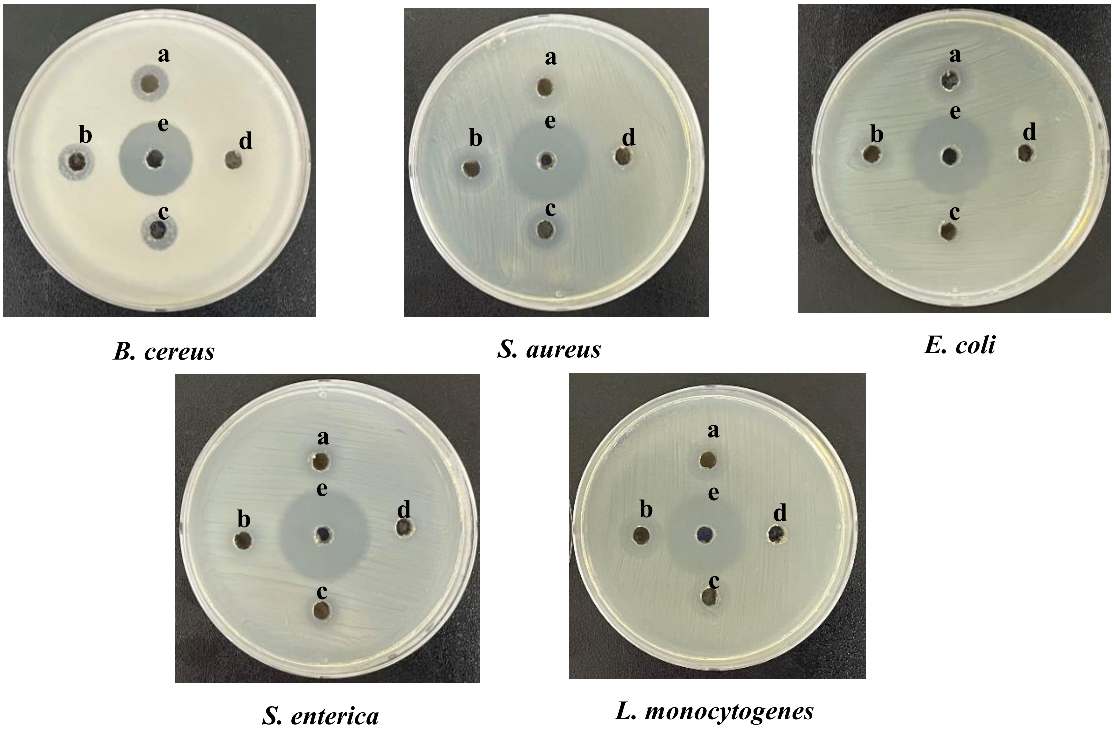

Figure S1. Antibacterial activity of S-AgNP NPs Nanoparticles. S-AgNP NPs with 0.5% starch (a); S-AgNP NPs with 1% starch (a); S-AgNP NPs with 1.5% starch (a); S-AgNP NPs with 2% starch (a); tetracycline hydrochloride (TCH; d).

Table S1. Antibacterial activity of S-AgNP NPs.

	Zone of inhibition (mm)			
	0.5 %<br>Starch<br>Ag NP NPs	1 %<br>Starch<br>Ag NP NPs	1.5%<br>Starch<br>Ag NP NPs	2 %<br>Starch<br>Ag NP NPs
S. aureus	4.80 ± 0.23^{a}	6.20 ± 0.23^{b}	7.30 ± 0.20^{c}	0.50 ± 0.02^{d}
S. enterica	3.67 ± 0.47^{b}	4.60 ± 0.22^{a}	5.20 ± 0.40^{a}	0.50 ± 0.02^{c}
L.monocytogenes	4.80 ± 0.40^{b}	4.60 ± 0.22^{b}	6.50 ± 0.41^{a}	0^{c}
E. coli	5.20 ± 0.23^{b}	6.00 ± 0.20^{a}	5.20 ± 0.40^{b}	0^{c}
B. cereus	6.20 ± 0.23^{b}	7.00 ± 0.00^{a}	7.10 ± 0.22^{a}	$6.67 \pm 0.46^{a,b}$

Values followed by the superscript letters in the column significantly differ ($p<0.05$)

(a)

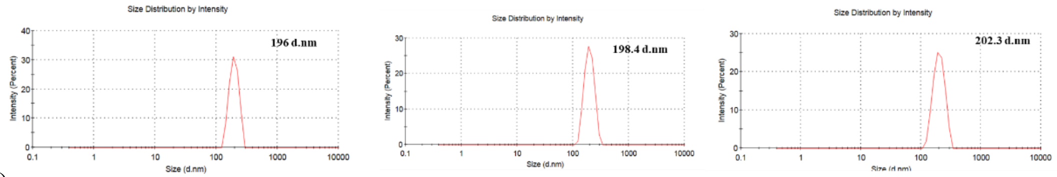

(b)

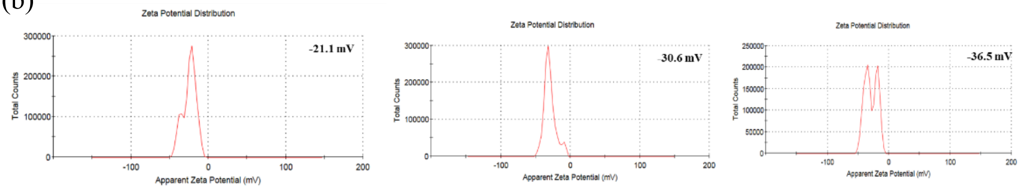

Figure S2. Hydrodynamic particle size (a) and zeta potential analysis (b) of S-AgNP NPs.

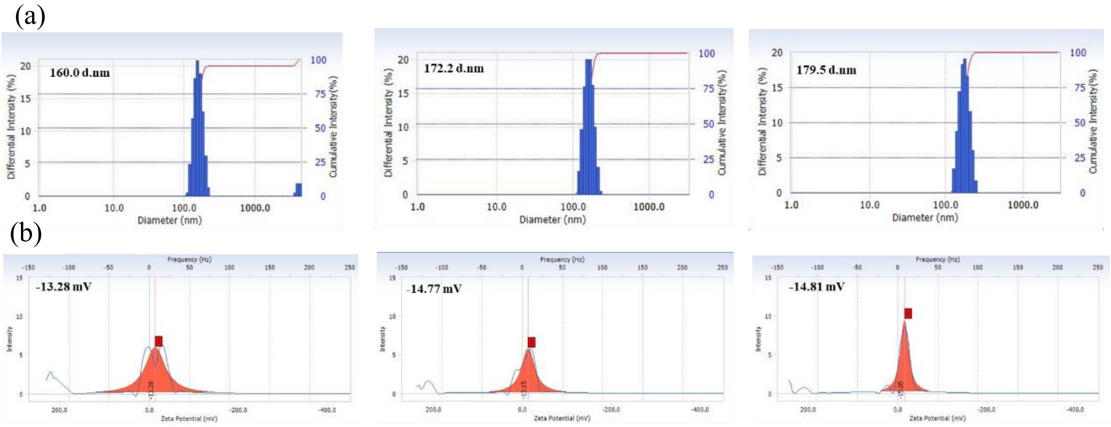

Figure S3. Hydrodynamic particle size (a) and zeta potential analysis (b) of AgNP NPs.

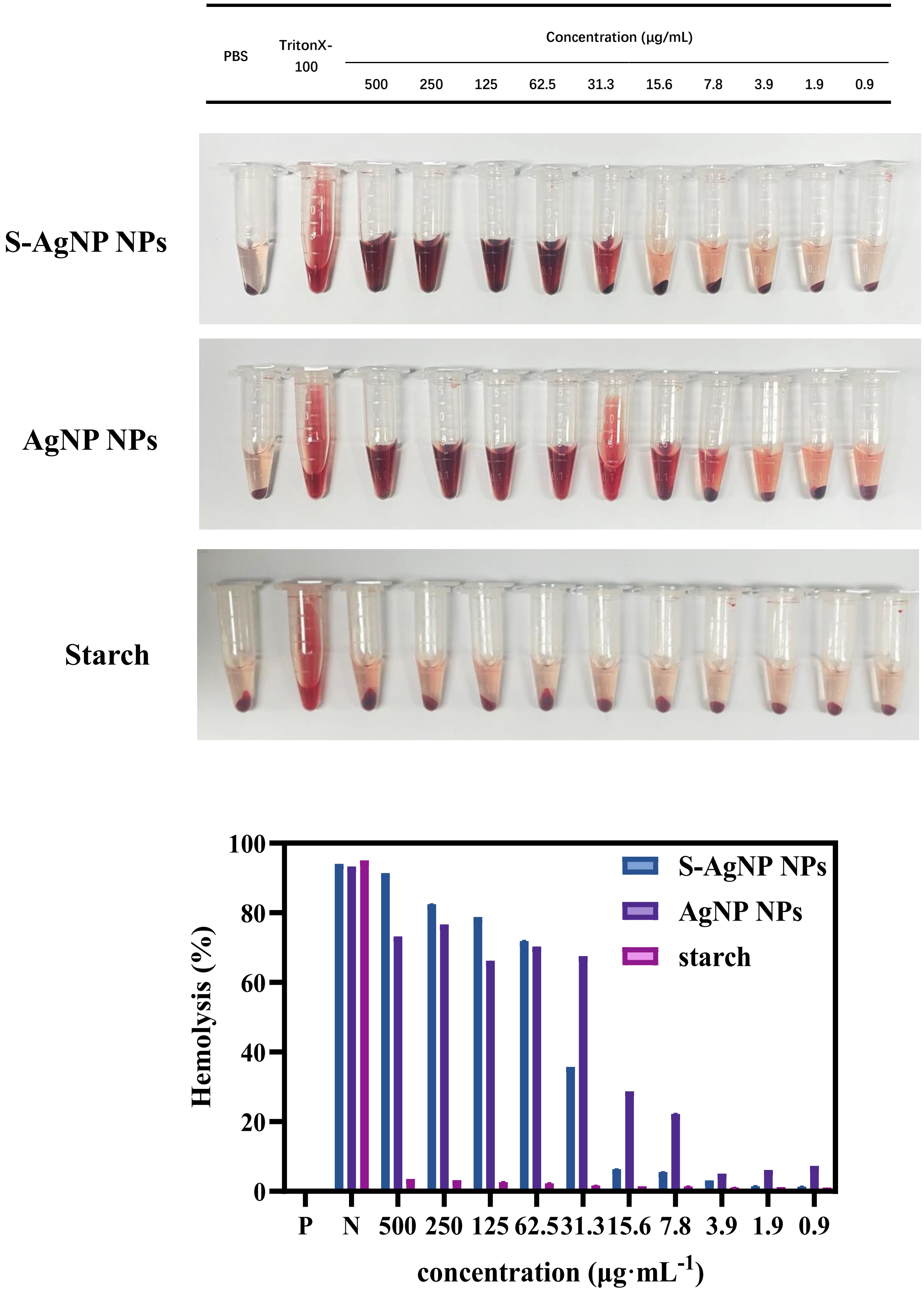

Figure S4. Hemolysis properties of S-AgNP NPs and AgNP NPs Incubated for 24 hours.
